# The role of oxidative stress in Friedreich's ataxia

**DOI:** 10.1002/1873-3468.12928

**Published:** 2017-12-20

**Authors:** Federica Lupoli, Tommaso Vannocci, Giovanni Longo, Neri Niccolai, Annalisa Pastore

**Affiliations:** ^1^ Department of Biotechnology, Chemistry and Pharmacy University of Siena Italy; ^2^ The Maurice Wohl Institute Dementia Research Centre King's College London UK; ^3^ Istituto di Struttura della Materia–CNR Rome Italy; ^4^ Department of Molecular Medicine University of Pavia Italy

**Keywords:** frataxin, mitochondrial diseases, neurodegeneration, oxidative stress, triplet

## Abstract

Oxidative stress and an increase in the levels of free radicals are important markers associated with several pathologies, including Alzheimer's disease, cancer and diabetes. Friedreich's ataxia (FRDA) is an excellent paradigmatic example of a disease in which oxidative stress plays an important, albeit incompletely understood, role. FRDA is a rare genetic neurodegenerative disease that involves the partial silencing of frataxin, a small mitochondrial protein that was completely overlooked before being linked to FRDA. More than 20 years later, we now know how important this protein is in terms of being an essential and vital part of the machinery that produces iron‐sulfur clusters in the cell. In this review, we revisit the most important steps that have brought us to our current understanding of the function of frataxin and its role in disease. We discuss the current hypotheses on the role of oxidative stress in FRDA and review some of the existing animal and cellular models. We also evaluate new techniques that can assist in the study of the disease mechanisms, as well as in our understanding of the interplay between primary and secondary phenotypes.

## Abbreviations

**CRISPR**, clustered regularly interspaced short palindromic repeat

**FRDA**, Friedreich's ataxia

**ROS**, reactive oxygen species

**SOD**, superoxide dismutase

Free radicals are molecules with free spared electrons that make the molecule highly reactive and thus dangerous. They are by‐products of normal cell function. The cell contains a number of mechanisms to absorb and neutralize them. However, when these mechanisms are overwhelmed or insufficient, free radicals can cause harm by inducing the oxidation of proteins and other essential molecules and causing damage 1. Free radicals can be generated by diet, stress, smoking, alcohol, exercise, inflammation, drugs, or exposure to the sun or air pollutants. Oxidative stress may contribute to the development of many diseases and chronic conditions, including cancer, neurodegeneration and diabetes. In this review, we focus on Friedreich's ataxia (FRDA), a rare mitochondrial neurodegenerative disease that constitutes an excellent example of a pathology associated with the presence of iron deposits and oxidative stress 2. We review the state of the field, paying particular attention to some of the animal and cellular models that have been developed with the aim of studying the role of free radicals and oxidative stress in this disease and we also suggest new strategies for studying their development with respect to disease aetiology and progression. Because the literature on FRDA is very abundant, we apologize to those colleagues whose work was not included in our review.

## Phenotype and genetic causes of Friedreich's ataxia

Patterns of one or more nucleotide repeats are common in genomes 3. These regions can be subjected to genomic instabilities that result in their expansion and, in some cases, cause disease. The vast majority of the related pathologies have neurological and/or developmental effects. FRDA is included among the neurological diseases that are a result of a triplet expansion. FRDA comprises an autosomal recessive disease that causes progressive damage to the nervous system resulting in symptoms ranging from gait disturbance and speech problems to heart disease. It was named after the physician Nikolaus Friedreich, who first described the condition to the medical community in 1863 4, 5, 6, 7. FRDA is characterized by a progressive degeneration of large sensory neurons and cardiomyopathies 8. Although rare, FRDA is the most frequent inherited ataxia, with an estimated prevalence of two to four people in 100 000 individuals and a carrier frequency of approximately 1 : 90 to 1 : 60 with a prevalence in white populations. Most FRDA carriers and affected FRDA patients are assumed to originate from a common European ancestor who lived more than 10 000 years ago 4, 9, 10. FRDA symptoms usually begin between the ages of 5 and 15 years but can, on rare occasions, appear as early as 18 months or as late as 50 years of age. The first symptom usually is gait or difficulty in walking. The ataxia gradually worsens and slowly spreads to the arms and then to the trunk. Sometimes, foot deformities may be early signs. Gradually, muscles begin to weaken and waste away, especially in the feet, lower legs and hands. Another symptom is the loss of tendon reflexes and, often, a gradual loss of sensation in the extremities, which may spread to other parts of the body. Rapid, rhythmic, involuntary movements of the eyes are common. Most FRDA patients develop scoliosis, which, if severe, may impair breathing and cause dysarthria, making the patients easily fatigued. Other symptoms that may occur include chest pain, shortness of breath and heart palpitations. These later symptoms are a consequence of the heart diseases associated with cardiomyopathy. Approximately 20% of people with FRDA develop carbohydrate intolerance and 10% develop diabetes mellitus. As a consequence, some people lose hearing or eyesight. The 20th anniversary of the discovery of the FRDA gene was in 2016 11. The gene was identified on chromosome 9q21.11 by positional cloning. It was demonstrated that FRDA is associated with a pathological expansion of a GAA‐TTC repeat in the first intron of the locus *X25*, later named *FXN* (HGNC: 3951), and is present in 98% of the affected alleles. A minority of patients are heterozygous for the expansion and mutations if the *FXN* gene. The expanded intronic alleles interfere with *FXN* transcription via epigenetic modifications, decreasing the production of the normally functioning *FXN* product, the frataxin protein, to 5–20% of the normal levels. The age of disease onset, severity, rate of progression and extent of neurological involvement all vary with the number of repetitive GAA sequences. The larger the number of repeats, the more profound is the reduction in frataxin expression and thus the disease symptoms. The critical pathologic triplet repeat threshold is 66 repeats, with the average expansion being as many as 630 GAA repeats on the smaller alleles and 890 GAA repeats on the larger ones. Frataxin is expressed in all cells of eukaryotic organisms. mRNA levels and frataxin expression have tissue specificities that partially correlate with the organs mostly affected by the disease. In humans, the highest levels of expression are found in the heart and spinal cord, whereas lower levels are seen in the cerebellum, liver, skeletal muscle and pancreas. The differential sensitivity of tissues to frataxin deficiency remains unclear, as do the epigenetic factors that determine the severity of the disease; it is not always the case the protein levels correlate with disease severity 12. FRDA patients are known to have oxidative stress and iron accumulation in mitochondria 13, 14.

## Searching for the frataxin function

**Figure 1:**
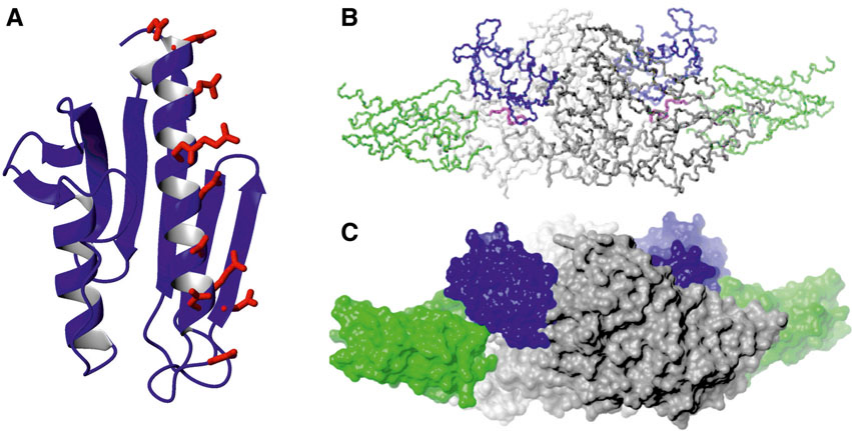
The structure of frataxins. (A) Ribbon representation of human frataxin (1ekg). The side chains of the residues of the exposed negatively‐charged ridge are explicitly shown. (B,C) Backbone and space filling representations of the complex of bacterial frataxin (CyaY) with the two central components of the iron‐sulfur cluster biogenesis, IscS and IscU 25.

Frataxin is a small (210 amino acids) protein localized in the inner mitochondrial membrane 11. It is synthesized in the cytosol as a precursor protein (1–210) and matured in two steps within the mitochondrial matrix to give an intermediate (42–210) and a mature form (81–210) 15. The structure of frataxin was first established by NMR spectroscopy for human frataxin (92–210) 16 (Fig. 1A). The crystal structures of human, yeast and *Escherichia coli* frataxins were also published 17, 18, 19, although that for yeast had to be revised more recently and was shown to be partially based on wrong spectral assignment 20. All of these structures share a similar fold, which directly reflects a high degree of sequence conservation and strongly suggests a common function. The fold of the conserved core consists of a globular, slightly elongated domain in which two N‐ and C‐terminal α helices pack against a central β sheet. Conservation throughout species indicates which residues are essential, either for folding or for function: conserved and semi‐conserved residues cluster onto the same surface 16. Frataxins are unusual iron‐binding proteins: they achieve iron coordination solely through exposed glutamates and aspartates clustered mostly on the first helix, instead of the more common cysteines or histidines 21, 22. CyaY and Yhf1, the bacterial and yeast frataxins, respectively, bind two Fe^2+^ ions 23. Additional weaker binding sites allow further loading of 25–26 cations per monomer. Another unusual property is the apparent lack of selectivity. CyaY and, to a minor extent, human frataxin are able to bind various divalent and trivalent cations, ranging from Ca^2+^ and Co^2+^ to Al^3+^ and some lanthanides 21. All of these cations compete for the same binding sites. Importantly, it is now established that frataxin is an active component of the iron‐sulfur cluster biogenesis machine, an essential metabolic pathway found in all organisms 24, 25. Direct interaction between frataxin and the NFS1/IscU complex, the two central components of the iron‐sulfur cluster biogenesis machine, was shown in several species (Fig. 1B,C) 26. The interaction was shown to result *in vitro* in a regulatory effect of frataxin on the rate of enzymatic formation of the clusters 24, 25. If true also *in vivo*, this role would explain the presence of iron accumulation in FRDA patients: insufficient levels of regulation would lead to an imbalance of iron which, when not being used for cluster formation, would precipitate, as seen in FRDA patients.

## The time dimension of FRDA

Consistent with its link as a regulator of the iron‐sulfur machine, from early on, FRDA was demonstrated to be associated with oxidative stress and reactive oxygen species (ROS) accumulation 14. Afterwards, this was also observed in yeast and mouse models 27, 28. However, an important aspect for understanding FRDA is the determination of the time evolution of this disease and when ROS start to accumulate. This is a theme of high general interest for all diseases: it is often difficult to distinguish between causes, effects and co‐existing phenomena in a disease. For example, one could consider whether a certain symptom (e.g. back pain) is what determines a headache or whether the two problems (i.e. headache and back pain) co‐occur by chance. Understanding this relationship is nevertheless essential because, in treatment, we would like to address directly the causes and not the side effects. FRDA does not escape this rule: despite having been studied extensively, it is still unclear whether oxidative stress is a primary cause or a secondary effect that occurs as a by‐product 29. The difficulty in answering to this question comes from the fact that patient samples, which are the primary source of our knowledge, have been exposed to the pathological condition for years since birth. When analyzed, they can only reveal an advanced state. Clarification can be achieved by developing inducible cellular or animal models that allow us to knockout, knockdown and overexpress the frataxin gene, starting from a well‐defined initial time point from which the phenotype can be induced. New tools have recently appeared to assist with this process and allow us to follow the causal progression of disease by genome editing and the production of complex cell models 26, 27, 28. They involve three main methodologies: (a) zinc finger nucleases 30; (b) the transcription activator‐like effector nuclease or TALEN 31; and (c) the clustered regularly interspaced short palindromic repeats (CRISPR) which, together with an RNA guided DNA endonuclease enzyme, appear to comprise a widely used frontier 32.

Different models of FRDA are now available in yeast, fly, worm, mouse and human cells, which we now discuss in some detail. As a result of the wide range of phenotypes found in the different models, we attempt to group the existing models by the animal/cell type. The main phenotypes found in the different cell/animal models are summarized in Table1.

**Table 1:** Summary of the animal/cellular models discussed in the present review

Animal models	Cellular phenotypes	Reference
Yeast models	Mitochondrial iron accumulation, oxidative stress, decreased Fe–S enzymes activity and oxygen consumption rate reduction	28, 33, 34, 35, 36
*Drosophila* models	Altered lipid synthesis with accumulation of lipid droplets, mitochondrial iron accumulation, decreased Fe–S enzymes activity, oxidative stress^a^, decreased ATP production	38, 39, 42, 43, 44, 45
Mouse models	Altered lipid synthesis, mitochondrial iron accumulation, decreased Fe–S enzymes activity, oxidative stress^b^	27, 48, 49, 50, 51, 52, 53

a Found in most models, except in Chen *et al*. 39.

b Found in Al‐Mahdawi *et al*. 27 and Poburski *et al*. 53.

## Yeast models

Yeast has been extensively used as a FRDA model as a result of being the most characterized eukaryotic cells and because of its simplicity as a cell model and the wide homology between human frataxin and the yeast orthologue, Yfh1. This simple organism is also particularly suited for temporal studies in which the effects of frataxin depletion on metabolism can be followed from the early phenotype onset onwards. One of the very first yeast models to take advantage of an inducible *YFH1* gene was developed by Radisky *et al*. 28. The pMETYFH yeast strain had knockout of the *YHF1* gene and carried an exogenous *YFH1* gene under the control of a methionine‐dependent promoter. This strain had a tightly regulated expression of frataxin with the ability to repress its expression in 2 h subsequent to the addition of methionine to the medium. Regulation of the inducible *YHF1* resulted in a direct effect on the *FET3* gene, a component of the high affinity iron uptake system of the plasma membrane. Repression of *YFH1* induced the up‐regulation of *FET3*, with increased uptake of iron by the cell followed by iron accumulation in mitochondria. By contrast, expression of the frataxin homologue induced a decrease in *FET3* expression. It was suggested that the accumulation effect was not the result of an increase in iron uptake by the mitochondria but, instead, the result of a reduction of efflux, the process that exports iron out of the mitochondria. Taken together, these data indicate a role of Yfh1 as a regulator of mitochondrial iron homeostasis. It was concluded that mitochondrial damage associated with *YFH1* repression was caused by an iron‐dependent increase in oxidative stress 28.

Ten years later, Moreno *et al*. 33 developed a refined inducible yeast model by modifying the promoter of the endogenous *YFH1* gene with a TetO promoter. This system allowed the Yfh1 expression to be repressed by adding doxycycline to the medium. The temporal relationship of different phenotypes was followed more accurately by analysing the effect of *YFH1* repression at several time points throughout a 72 h window. Iron accumulation in mitochondria was the first phenotype to appear (14 h) followed by a decrease of aconitase and its activity. Following the respiratory chain proteins (complexes I and III), which are part of another metabolic pathway strictly associated with iron‐sulfur cluster proteins, the oxygen consumption rate, as a measure of the respiratory chain activity, started to decrease only after iron accumulation. The expression levels of *FET3* were found to be similar to those observed by Radisky *et al*. 28 and Moreno *et al*. 33. In agreement with previous studies in which a reduction of superoxide dismutase (SOD) activity in Δ*YFH1* cells 34, 35 and an increase in carbonylated proteins 36 were observed, protein carbonylation was detected by Moreno *et al*. 33, although only 24 h after the repression of *YFH1*. This occurs clearly after the detection of iron deposits and more or less at the same time as the reduction in aconitase activity. SOD activity showed a steady decrease with a significant decline after 24 h. These results all suggested an iron‐dependent increase in ROS that affects iron‐sulfur cluster containing proteins. Aconitase activity in *YFH1* cells could be preserved only under strict anaerobic conditions 36, indicating a correlation between its reduction and ROS increase rather than a link with reduced iron‐sulfur cluster biogenesis. This model was used to suggest a correlation between disruption of iron homeostasis and the metabolic reprogramming that would induce a reduction in iron‐sulfur cluster biogenesis 37.

## *Drosophila* models

Navarro *et al*. 38 produced a *Drosophila* model based on RNA interference in 2010 that showed the accumulation of lipid droplets in glial cells, lipid peroxidation, increased susceptibility to oxidative stress, neurodegeneration and a reduced life span. It was concluded that lipid accumulation was either the result of an increase in synthesis or a reduction in lipid catabolism. Some of the detected phenotypes were ameliorated by overexpression of the ApoD homologue *GLaz*, a protein involved in the lipid metabolism that has a role in oxidative stress defences of fly cells. The accumulation of lipid droplets was one of the main phenotypes detected in a second *Drosophila* model developed by Chen *et al*. 39 in 2016. This model was based on a mosaic mutant with *fh* photoreceptor neurons. Young flies showed an expansion of the endoplasmatic reticulum and an accumulation of lipid droplets, whereas degeneration of the photoreceptor was subsequently detected in older flies. It was also observed that mitochondria had abnormal morphology, complex I was compromised and ATP production severely reduced. *Fh* knockout was strongly associated with iron accumulation in mitochondria but, as opposed to the results found by Navarro *et al*. 38, it was not possible to detect any increase in oxidative stress and overexpression of the ROS scavengers SOD1 and SOD2 did not reduce neurodegeneration, indicating a ROS‐independent mechanism. A connection was suggested between iron accumulation and increased sphingolipid synthesis that, in turn, would activate Pdk1, a kinase, and Mef2, a transcription factor associated with muscle differentiation, leading to neurodegeneration 39. A ROS‐independent neurodegeneration mechanism in *Drosophila* 40, 41 was, however, not supported by all models. An increased sensitivity to H_2_O_2_ was shown in an RNA interference model 42. Interestingly, mortality in flies was rescued by the overexpression of CAT, a peroxisomal catalase with ROS protective properties, but not by overexpression of SOD1 and SOD2 42. Treatments with the antioxidant idebenone or with rapamycin were also shown to produce a protective effect against oxidative stress in two additional *Drosophila* models 43, 44. The role of oxidative stress in an RNA interference *Drosophila* model was also demonstrated in a different study showing that downregulation of *fh* and hyperoxia conditions greatly affect aconitase activity and reduce the life span of flies 45. Reduction of aconitase activity was only detected when the flies where subjected to hyperoxia condition, suggesting a protective role of the frataxin orthologue against oxidative stress. On the other hand, increased oxidative stress did not affect the activity of succinate dehydrogenase, suggesting that this effect did not extend to all iron‐sulfur cluster enzymes 45.

## Mice models

One of the very first models of FRDA was developed in mouse leading to complete knockout. This model resulted in embryonal death demonstrating the essential nature of the *Fxn* gene 46. Subsequently, Ristow *et al*. 47 developed a mouse cell model in which they induced frataxin overexpression and observed a calcium‐induced upregulation of the tricarboxylic acid cycle flux and respiration, which resulted in an overall increase of the cellular ATP levels. These results suggested a role of frataxin in mitochondrial energy conversion and oxidative phosphorylation but provided little information about the temporal appearance of oxidative stress 47. Chen *et al*. 48 developed a knockout *Fxn* mouse model using CRISPR/Cas9 in an attempt to reproduce the results obtained in their *Drosophila* model 36. The gene editing molecules were delivered to the brain of young mice via adeno‐associated virus particles. The knockout mice showed a phenotype with a shorter life span, neurological damage and altered sphingolipid synthesis, in agreement with an earlier mouse model 49. Iron levels in the cortex were increased and the genes for PDK1 and Mef2 were up‐regulated. As previously reported in the fly model, Chen *et al*. 48 could not detect increase in oxidative stress (lipid peroxidation) 48. The two models in *Drosophila* and mouse also agreed with the data obtained from FRDA patient samples where PDK1 and sphingolipids were found to be increased. These results indicated a consistency between different organisms and were in agreement with a liver knockout mouse model in which abnormal lipid metabolism was detected in the form of accumulated lipid droplets 50. This phenotype was detected at an early stage (4 weeks) and was associated with abnormal mitochondria, iron‐sulfur cluster biogenesis disruption and, in some cases, electron‐dense structures typical of iron deposits. Regarding the model by Chen *et al*. 48, another mouse model (Frda/MCK mouse) suggested an oxidative stress‐independent mechanism 51, 52. The primary phenotype identified by Seznec *et al*. 51 was a decrease in iron‐sulfur cluster enzyme activity followed by iron accumulation. Oxidative stress did not appear to be part of the pathophysiology and, accordingly, approaches to increase antioxidant defences had no effects 51.

These models all shared disruption of iron‐sulfur cluster biogenesis, oxidative stress and iron accumulation, which are typical of FRDA patients. However, the results obtained from different mouse models did not always agree on the temporal relationship between the phenotypes. A Cre/Lox inducible *Fxn* knockout model based on murine fibroblasts was recently used to address these discrepancies 53. The earliest event identified was a 14% reduction in aconitase activity followed by a decrease of ATP production and oxygen consumption. Oxidative stress, identified as an increase in ROS, was detected only after iron‐sulfur cluster disruption; iron accumulation was observed as a late event 53.

Conditional *Fxn* KO was not the only approach used to create a FRDA mouse model. A successful approach was based on the introduction of a human *FXN* gene construct in *Fxn* null mice. Several iterations of this model were suggested based on different GAA expansion lengths 27, 54. Characterization of the YG8 and YG8sR models, carrying *FXN* exogenous genes with 90 + 190 or 200 GAA repeats, respectively, showed age‐dependent FRDA symptoms such as ambulatory difficulties, decreased frataxin mRNA levels, abnormal root ganglia, reduced aconitase activity and oxidative stress 27, 54. Neurons derived from the YG8 mouse also showed a reduction in Complex I activity, increased oxidative stress in both mitochondria and cytosol, and lipid peroxidation 55. These models, in addition to recapitulating the characteristic FRDA phenotype, were also suitable for studying the genetic aspects of the disease, such as GAA repeat instability 56, gene silencing induced by the expansion 57 and novel gene therapy approaches 58, 59.

## A human cell model

In 2015, we developed a CRISPR‐based system and engineered a cell line based on immortalized human embryonic kidney cells, HEK293, in which an exogenous inducible *FXN* gene rescues the cells from biallelic knockout of the endogenous *FXN* genes 60. Even though this line may not be optimal for recapitulating the tissues mainly affected in FRDA patients, it was a convenient choice for establishing the proof of principle of the approach. The specific CRISPR used was chosen with respect to the required proximity of its target sequence to exon 4 of *FXN*. We obtained a targeting construct (pFSVpur‐LoxP‐TCI4) which, when integrated by homologous recombination, was able to excise exon 4 completely and replace it with a puromycin resistance cassette. We then produced knockout of both *FXN* alleles, which required two rounds of transfection with CRISPR‐I4 and the targeting construct because simultaneous homozygous *FXN* knockout is a rare event. The presence of the puromycin cassette flanked by two Lox‐P sites allowed us to select the targeted cells in the first round, followed by Cre recombinase‐mediated excision of the puromycin cassette and a second round of targeting using the same pFSVpur‐LoxP‐TC‐I4 construct. The targeting experiments carried out with CRISPR‐I4 and pFSVpur‐LoxP‐TC‐I4 showed a targeting frequency of ~ 50% compared to a frequency of 0% when cells were transfected with only a pFSVpur‐LoxP‐TC‐I4 targeting construct. This step therefore proved the feasibility of successfully performing gene editing at the *FXN* locus. The inducible *FXN* cassette allowed us to modulate the amount of frataxin in the cell by over or under‐expression of the gene itself. This system allowed us not only to gain insights into the disease mechanism (under‐expression), but also to obtain useful information on the effects of frataxin over‐expression on several mitochondrial pathways using a number of traditional and new biomarkers that detect cellular ROS, along with indicators of iron‐sulfur cluster formation such as aconitase levels, as successfully used in other FRDA studies 61. We discuss the different approaches below. In the future, we aim to develop a new cellular model that mimics more faithfully the tissues affected by FRDA based on inducible pluripotent stem cells. These cells have the advantage of differentiating in FRDA‐relevant cell types such as sensory neurons and cardiomyocytes, as well as having a normal karyotype (normal diploid, normal XY). Additionally, compared to previous inducible pluripotent stem cells derived from FRDA patients, this novel cell model will allow us to study the very early phenotype of the disease.

## New methodologies to the study of oxidative stress

Because of the heated debate on the role of oxidative stress in FRDA, it is important to develop new technologies that allow us to detect its early occurrence and to follow up its development. Most of the early studies of FRDA used the activity of aconitase and other mitochondrial enzymes as a parameter to follow the phenotype 62. This important parameter, however, may be insufficient to accurately describe the disease progression. More recently, other markers have been used. The development of new fluorescent probes can allow a sensitive quantification of ROS 63, 64. The introduction on the market of the Seahorse XF technology (Mito stress test; Agilent Technologies Inc., Santa Clara, CA, USA) has allowed the direct measurement of the oxygen consumption rate in living cells, which represents a very important parameter for understanding the degree of cellular stress 65. In association with three different compounds (oligomycin, carbonyl cyanide p‐(trifluoromethoxy)‐phenylhydrazon and a mixture of rotenone/antimycin A) that are sequentially added to the medium, the measured variation in oxygen consumption rate can be used to assess, simultaneously, the state of several mitochondrial functions, such as basal respiration, ATP production, maximal respiration and non‐mitochondrial respiration. Another technique that we recently developed for the study of FRDA is in‐cell infrared absorption spectroscopy, which provides valuable information on the structure content of cellular components associated with correlated cellular microscopy analysis. Correlated cellular microscopy relies on two‐dimensional correlation spectroscopy 66 to assign complex band patterns in cellular spectra, based on the correlation of their changes and the clustering together of the bands that evolve in synchrony over time. Finally, we propose the possible application of a novel promising biosensor, the nanomotion sensor, for performing real‐time, correlated measurements of different cellular nanoscale biomotions and metabolic activities, at the same time as stimulating the cells with physical or chemical stimuli 67. This innovative method combines conventional bio‐investigation techniques and custom analysis chambers 68 with a nanomechanical oscillator, typically an atomic force microscopy cantilever, leading to a device that can transduce the smallest cellular motion or vibration in measurable signals 69. The resulting electrical signal yields real‐time information on the metabolic state of cells incubated on the cantilever in the analysis chamber. Together, these old and new techniques could provide new and complementary information and help us to reconstruct the disease progression of FRDA.

## Conclusions

In conclusion, in the present review, we have discussed how FRDA can be considered as a prototypical example of the problems associated with ROS increase and oxidative stress, as well as the solutions that have been proposed to study this disease. The models suggested have provided useful indications that can, however, lead to different conclusions. Their discrepancies are likely the consequence of the nature of the chosen cell/organism, the specific model and the biomarkers used. Another important factor that could explain the different responses is the time frame over which the phenotype progression is followed: for example, this was 0–72 h in a yeast model 33, 0–12 days in *Drosophila* 39 and 0–10 days in mice 53. We consider that most of the methodologies developed for FRDA can also be applied successfully to study other diseases in a time‐resolved way, which, in the future, will allow us to better place the role of oxidative stress in disease.
